# Ameliorative Effects of Vitamin E and Lutein on Hydrogen Peroxide-Triggered Oxidative Cytotoxicity via Combined Transcriptome and Metabolome Analysis

**DOI:** 10.3390/cells14242020

**Published:** 2025-12-18

**Authors:** Hongrui Lv, Yongji He, Shang Guo

**Affiliations:** Shanxi Institute for Functional Food, Shanxi Agricultural University, Taiyuan 030031, China

**Keywords:** vitamin E, lutein, ameliorative effects, oxidative stress, transcriptome, metabolome

## Abstract

Vitamin E and lutein both belong to food functional factors, which have cytoprotective potential and antioxidant effects. However, mechanism details at cell level remain scarce. In this study, HepG2 cells were utilized to inquire and compare the ameliorative effects of vitamin E and lutein under H_2_O_2_-induced oxidative stress through a combined transcriptomic and metabolomic profiling, in addition to physiology and biochemistry determination. Cell cytotoxicity caused by H_2_O_2_ was ameliorated by vitamin E or lutein as evidenced by elevating cell viability and balancing the redox system. Vitamin E had greater efficacy on ameliorating oxidative cytotoxicity than lutein. Transcriptome data revealed that differentially expressed genes were mainly enriched in the transport-related, enzyme-related, and oxidative stress-related GO terms with vitamin E pretreatment. Extracellular organization-related, biological process-related, and apoptosis-related GO terms were meaningfully enriched with lutein pretreatment. Metabolome data showed that with vitamin E ameliorative effects, the disturbed metabolic pathways included thiamine metabolism, vitamin digestion and absorption, and ABC transporters. With lutein ameliorative effects, KEGG pathway analysis showed enrichment of amino sugar and nucleotide sugar metabolism, pyrimidine metabolism, and starch and sucrose metabolism. Collectively, our study provides essential insights into utilization of vitamin E and lutein as a potential supplement for effective therapy of disease associated with oxidative stress.

## 1. Introduction

Oxidative stress is implicated in the production of intracellular reactive oxygen species (ROS), which overcome cells’ ability to scavenge them, thereby disrupting the redox balance [1]. ROS consists of various reactive chemical molecules including hydroxyl radicals, superoxide anions, and hydrogen peroxide (HP; H_2_O_2_), which are associated with orchestration of cellular proliferation, apoptosis, signaling, and immunity [2]. ROS are primarily produced by cytochrome P450 enzymes in hepatocytes, and the major sites are mitochondria and endoplasmic reticulum [3]. Liver is a primary organ associated with detoxification metabolism and is especially particular to the negative impact by surplus ROS [4]. Oxidative stress induced by unnecessary ROS is closely related to several chronic diseases, covering atherosclerosis, neurodegenerative disorders, hyperglycemia, cancer, and cardiovascular disease [5]. Therefore, protection against surplus ROS is crucial to maintain overall health.

Some food functional factors could mitigate the negative impact caused by free radicals. As is well-known, vitamin E is a fat-soluble micronutrient obtaining through diet, and it belongs to non-enzymatic antioxidants. During lipid peroxidation process, vitamin E could supply hydrogen to eliminate oxygen free radicals [6]. Vitamin E could promote cardiovascular health and strengthen the antioxidant defenses of low-density lipoproteins [7]. Vitamin E holds promise in mitigating oxidative stress-related disease [8]. Lutein is a ubiquitous carotenoid in green vegetables, which contributes to eye protection due to its inherent antioxidative and light absorption properties [9]. The benefits of lutein also extend to immune system modulation and intracellular antioxidant improvement [10].

However, the antioxidant mechanisms of vitamin E and lutein were partially studied, and at the same time, the comprehensive whole-molecular study is limited. The combination of emerging “omics” technologies like transcriptomics and metabolomics provides a powerful suite for uncovering key genes, metabolites, and underlying mechanisms in response to interventions, offering novel insights into complex biological networks [11]. To our knowledge, the beneficial effects of vitamin E and lutein stayed at the physiological level, and the comparison study using combined “omics” approaches remains scarce. Hepatocellular carcinoma cell lines (HepG2 cells) are widely utilized as an in vitro liver model, and HP produced from almost all source of oxidative stress, which are both widespread used for oxidative resistance research [4]. In the study, HepG2 cells were employed to novelly compare the transcriptome and metabolome changes induced by vitamin E (nutritional ingredient) and lutein (bioactive substance) under HP-induced oxidative stress. Along with physiology and biochemistry measurements, the study highlights the promise of food functional factors for the therapeutic application against oxidative stress-related diseases.

## 2. Materials and Methods

### 2.1. Cell Culture and Manipulations

HepG2 cells, obtained from Wuhan Pricella Biotechnology Co., Ltd. (Wuhan, China), were maintained in Dulbecco’s Modified Eagle’s Medium (DMEM; Gibco, Waltham, MA, USA) containing 10% fetal bovine serum (Pricella, Wuhan, China) and 1% penicillin/streptomycin (Pricella, Wuhan, China) in an incubator filled with 5% CO_2_ at 37 °C. Vitamin E and lutein were supplied by TargetMol Chemicals Inc. (Boston, MA, USA). HepG2 cells were inoculated in appropriately cell culture bottles or plates for adherent growing, and subjected to vitamin E, lutein, or HP treatment. The ameliorative effects of vitamin E and lutein pretreatment against HP-triggered oxidative cytotoxicity was investigated and compared. Each experiment was repeated six times.

### 2.2. Cell Viability Assay

HepG2 cells were inoculated in 96-well plates with 1 × 10^4^ cells per well and allowed to adhere for 12 h. Then, cells were exposed to various concentrations of vitamin E and lutein for 12 h for preliminary examination of the compounds cytotoxicity without stressor, which indicated vitamin E and lutein had no cytotoxicity up to 20 μM and 120 μM, respectively (Figure S1). According to the preliminary results, cells were exposed to low, medium, or high concentrations (relative to toxic concentration) of vitamin E (5, 10, 20 μM) or lutein (40, 80, 120 μM), followed by a 12 h incubation. Finally, HP ranging from 0.1 mM to 2.0 mM were used to induce oxidative stress for 6 h.

Cell viability was evaluated utilizing the Cell Counting Kit-8 (CCK-8; Bioss, Beijing, China) in accordance with the manufacturer’s protocol. The calculation was performed as follows: viability (%) = [(ODtreated − ODblank)/(ODcontrol − ODblank] × 100%. In addition, the optimal concentrations of vitamin E, lutein, and HP were determined based on cell viability results for subsequent experiments.

### 2.3. ROS Assay

The ROS was evaluated employing Reactive Oxygen Species Assay Kit (Beyotime, Shanghai, China), on the basis of 2′,7′-dichlorofluorescein diacetate (DCFH-DA) probe. DCFH-DA is hydrolyzed to DCFH when entering cell intra through the cell membrane, which is subsequently oxidized to the intense green fluorescent compound DCF by intracellular ROS [12]. Specifically, the cells measuring 1 × 10^4^ per well were inoculated in black 96-well plates and exposed to vitamin E, lutein, or HP at various concentrations. Afterwards, cells were loaded on 10 µM DCFH-DA, then incubated for 20 min at 37 °C. Fluorescence measurement was achieved through a multimode plate reader (BioTek, Winooski, VT, USA) at 488 nm excitation and 525 nm emission.

### 2.4. Antioxidant Measurements

Cells were exposed to optimal concentrations of vitamin E, lutein, or HP. We determined superoxide dismutase (SOD) and catalase (CAT) activities, as well as glutathione (GSH) levels employing assay kits obtained from Jiancheng Biotechnology Co. (Nanjing, China). The SOD activity was measured utilizing the water-soluble tetrazolium salt (WST-1) method based on the reaction between superoxide anion and WST-1 [13]. One unit of SOD activity inhibited 50% formation of WST-1. The CAT activity was investigated utilizing ammonium molybdate method. A yellow complex was formed from the reaction between H_2_O_2_ and ammonium molybdate, and the absorbance was quantified at 405 nm [14]. One unit of CAT activity denoted decomposing 1 μmol H_2_O_2_ per second. GSH level was determined at 405 nm on account of the reaction with 5,5′-dithiobis-(2-nitrobenzoic acid) to yield a yellow product [15].

### 2.5. RNA Extraction

A total of 5 × 10^6^ cells were collected from the CT, HT, ET, and LT groups with 6 replications. Total RNA was extracted employing TRIzol Reagent (Life Technologies, Carlsbad, CA, USA). RNA concentration and purity were determined utilizing NanoDrop 2000 (Thermo Fisher Scientific, Wilmington, DE, USA), while integrity was evaluated utilizing the RNA Nano 6000 Assay Kit of the Agilent Bioanalyzer 2100 system (Agilent Technologies, Santa Clara, CA, USA).

### 2.6. Transcriptome Sample Preparation, Library Construction, and Sequencing

An amount of 1 μg of total RNA from each sample served as the input material for preparing RNA samples. Sequencing libraries were generated by means of Hieff NGS Ultima Dual-mode mRNA Library Prep Kit for Illumina (Yeasen, Shanghai, China). Unique index codes were assigned to the sequences from each sample for subsequent identification. mRNA purification was achieved by poly-T oligo-attached magnetic beads. After double-stranded cDNA synthesis, the DNA fragments were blunt-ended by means of exonuclease/polymerase, adenylated at the 3′ ends, and ligated to NEBNext hairpin adaptors in preparation for hybridization. The library fragments were purified by AMPure XP system (Beckman Coulter, Beverly, MA, USA). Following size selection and adaptor ligation, the cDNA was then subjected to USER Enzyme (NEB, Ipswich, MA, USA) treatment (3 μL) at 37 °C for 15 min, followed by 95 °C for 5 min. PCR was then proceeded utilizing Phusion High-Fidelity DNA polymerase, employing both Universal and Index (X) primers. PCR products purification was conducted on AMPure XP system. Libraries quality assessment was performed on the Agilent Bioanalyzer 2100 system. The libraries were subjected to sequencing on an Illumina NovaSeq platform (Illumina, San Diego, CA, USA) to produce 150 bp paired-end reads.

### 2.7. Transcriptome Data Analysis

The raw reads were subjected to bioinformatic analysis utilizing BMKCloud (www.biocloud.net, accessed on 15 October 2025) platform. Raw FASTQ data were initially processed employing in-house Perl scripts, which served to remove adapter sequences and low-quality reads, and in parallel, generated Q20, Q30, GC-content, and sequence duplication level. Following data processing, raw sequences were converted to clean reads. Alignment of the clean reads against the reference genome was performed employing the Hisat2 software 2.0.4. The subsequent analysis was restricted to reads that aligned with either a perfect match or one mismatch for final annotation. Functional annotations for genes were derived from Gene Ontology (GO) databases. We quantified gene expression in fragments per kilobase of transcript per million fragments mapped (FPKM) and performed differential expression analysis between the two groups using DESeq2 1.30.1, which relies on a negative binomial model. Significant results were determined by applying a false discovery rate (FDR) adjustment to the *p* values on account of Benjamini and Hochberg’s approach. Differentially expressed genes (DEGs) were screened by the threshold of |log2Fold Change| ≥ 1.5 and FDR < 0.05.

### 2.8. Quantitative Real-Time Polymerase Chain Reaction (qRT-PCR) Validation

Expression changes in six target genes between four different treatment samples were validated by qRT-PCR. Total RNA was isolated utilizing Trizol reagent (Invitrogen, Carlsbad, CA, USA) and subsequently reverse-transcribed into cDNA employing the TUREscript 1st Stand cDNA SYNTHESIS Kit (Aidlab, Beijing, China). Subsequently, qRT-PCR proceeded on a Quantitative Real-Time PCR system (qTOWER, Analytik Jena AG, Jena, Germany) employing the 2×SYBR^®^ Green Master Mix (Takara, Beijing, China). We calculated the relative gene expression via the 2^−ΔΔCt^ method [16], using β-Actin for normalization. The corresponding primer sequences were provided in Table S1.

### 2.9. Metabolites Extraction

We transferred the sample each of 5 × 10^6^ cells from the CM, HM, EM, and LM groups with 6 replications to a 1.5 mL microcentrifuge tube by adding 1000 μL of an extraction solution (methanol: acetonitrile = 1:1 *v*/*v*) spiked with an internal standard (20 mg/L) in three sequential steps (300 μL, 300 μL, and 400 μL). We vortexed the samples for 30 s in an ice-water bath and homogenized them at 45 Hz for 10 min. Following incubating at −20 °C for 1 h, we centrifuged them at 12,000 rpm for 15 min at 4 °C to pellet the debris. A 500 μL aliquot of the resulting supernatant was carefully dispensed into a microcentrifuge tube and dried in a vacuum concentrator, which was reconstituted in 160 μL of an extraction solution (acetonitrile/water = 1:1, *v*/*v*), and sonicated for 10 min in an ice-water bath. Once again, the samples were centrifuged under the same conditions, and 120 μL of the supernatant was dispensed into a 2 mL sample vial. For quality control (QC), 10 μL aliquots from each sample were pooled to create a QC sample.

### 2.10. Ultra-High Performance Liquid Chromatography-Quadrupole Time-of-Flight-Tandem Mass Spectroscopy (UPLC-QTOF-MS/MS) Analysis

Metabolomic analysis was proceeded on a UPLC-MS/MS system composed of a Waters Acquity I-Class PLUS UPLC linked to a Waters Xevo G2-XS QTOF (Waters, Milford, MA, USA) high-resolution mass spectrometer. Fractionation was achieved using a Waters Acquity UPLC HSS T3 column (1.8 μm, 2.1 × 100 mm; Waters, Milford, MA, USA) with mobile phase of (A) 0.1% formic acid in water and (B) 0.1% formic acid in acetonitrile.

The Waters Xevo G2-XS QTOF high-resolution mass spectrometer was operated in MSE mode under MassLynx V4.2 control to acquire both primary and secondary MS data. Each acquisition cycle simultaneously collected data in two channels: one at low collision energy (switched off) and the other at a high collision energy ramp of 10–40 V, with a scan rate of 0.2 s per spectrum. The electrospray ionization source was configured with capillary voltages of 2500 V (positive) and −2000 V (negative), a cone voltage of 30 V, and temperatures of 100 °C (source) and 500 °C (desolvation). The backflush and desolvation gas flows were maintained at 50 L/h and 800 L/h, respectively.

### 2.11. Metabolome Data Analysis

Raw data from MassLynx V4.2 was subjected to Progenesis QI software 4.0 to facilitate metabolite identification. This involved peak extraction and alignment, followed by database searching against both the online METLIN and a self-built library. For metabolite quantification, the quadrupole filters the precursor ions of the target substance and excludes the ions corresponding to other molecular weights to eliminate interference. After obtaining the metabolite MS data, peak area integration was performed, which was used to determine the relative metabolite contents. Following normalization of the original peak areas to the total peak area, the annotated compounds were classified and mapped to Kyoto Encyclopedia of Genes and Genomes (KEGG) pathways. On the basis of group information, fold changes (FCs) were calculated, and the statistical significance *p* value was derived from T test for each compound. We constructed an orthogonal partial least squares-discriminant analysis (OPLS-DA) model with the ropls R package 1.6.2 and evaluated its reliability using 200 permutation tests. The variable importance in projection (VIP) values was determined by employing multiple cross-validation during the model fitting process. Differential accumulated metabolites (DAMs) were filtered on account of an integrated criteria FC > 1, *p* < 0.05 and VIP ≥ 1. We used hypergeometric distribution test to determine the KEGG pathways that were significantly enriched with DAMs.

### 2.12. Statistical Analysis

Data from six replicate experiments (n = 6) are calculated as mean ± standard deviation. We analyzed the data using SPSS 18.0 software. For multiple comparisons, one-way analysis of variance (ANOVA) was employed in conjunction with Dunnett’s test. Statistical significance was set at *p* < 0.05. Results are marked with different letters to denote statistical significance.

## 3. Results

### 3.1. HP Caused HepG2 Cell Cytotoxicity

**Figure 1:**
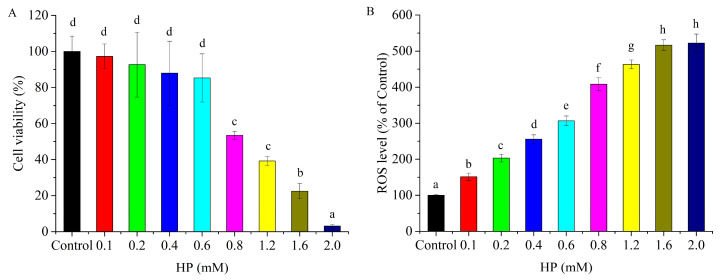
Cell cytotoxicity induced by H_2_O_2_. (**A**) Cell viability changes treated with different concentrations of H_2_O_2_ (0.1, 0.2, 0.4, 0.6, 0.8, 1.2, 1.6, 2.0 mM); (**B**) ROS accumulation exposed to different concentrations of H_2_O_2_. Different letters denote statistical significance.

HepG2 cells were exposed to HP (0.1, 0.2, 0.4, 0.6, 0.8, 1.2, 1.6, and 2.0 mM) for 6 h, and the cell viability was evaluated through the CCK-8 method. At lower concentrations (0.1–0.6 mM), no significant effect on HepG2 cell viability was observed. In contrast, exposure to 0.8–2.0 mM resulted in a statistically significant decline in viability (Figure 1A). 0.8 mM HP caused 50% decrease in cell viability, which was selected for further investigation. ROS accumulation leads to cellular damage and breaks the balance of redox system. With the increase in HP concentration, the ROS level is significantly upregulated. Compared with control group, ROS accumulation in 0.8 mM HP group rose by four times (Figure 1B).

### 3.2. Effects of Vitamin E and Lutein on HepG2 Cell Cytotoxicity

**Figure 2:**
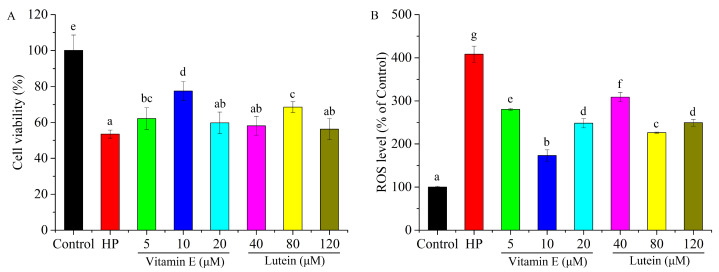
Effects of vitamin E and lutein on HepG2 cell oxidative cytotoxicity. (**A**) Ameliorative effects of vitamin E and lutein at different concentrations on cell viability under 0.8 mM HP-triggered oxidative stress. (**B**) Ameliorative effects of vitamin E and lutein at different concentrations on ROS level under 0.8 mM HP-triggered oxidative stress. Different letters denote statistical significance.

With pretreatment of different doses of vitamin E or lutein, the cell viability and ROS accumulation in HepG2 cells recovered in various degrees under oxidative stress. Concentrations of 5 and 10 μM vitamin E significantly improved the cell viability, whereas no significant change was observed at the concentration of 20 μM. Low (40 μM) and high (120 μM) concentrations of lutein have no effect on cell viability under HP treatment, whereas 80 μM lutein significantly relieved cell cytotoxicity (Figure 2A). Analogously, 20 μM vitamin E and 80 μM lutein alleviated HP-induced oxidative stress to the greatest extent, which was used for further investigation (Figure 2B). And we also found that vitamin E was more effective than lutein. The results suggested that treatment with vitamin E and lutein defended HepG2 cells from HP-induced cell cytotoxicity via decreasing the level of ROS.

### 3.3. Effects of Vitamin E and Lutein on Antioxidant System of HepG2 Cell Under Oxidative Stress

**Figure 3:**
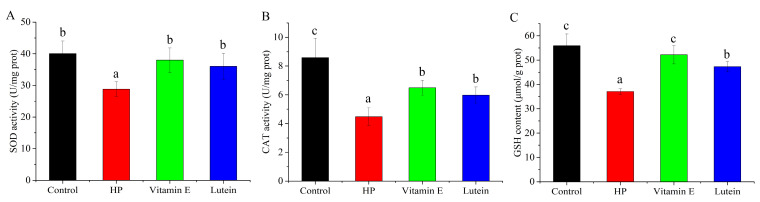
Effects of vitamin E and lutein on antioxidant system of HepG2 cells under oxidative stress. (**A**–**C**) 10 μΜ vitamin E and 80 μΜ lutein improved SOD activities, CAT activities, and GSH contents under 0.8 mM HP-triggered oxidative stress. Different letters denote statistical significance.

Antioxidant enzymes and non-enzymatic antioxidant play important roles in scavenging ROS to protect cells from oxidative stress. Compared with control group, the SOD, CAT activities and GSH content were markedly declined in HP-induced group. With pretreatment of vitamin E or lutein, SOD activity recovered to the control level (Figure 3A), and CAT activity significantly elevated (Figure 3B). GSH content in vitamin E treatment group returned to control level, and lutein was also able to significantly increase the GSH content (Figure 3C). The results revealed that pretreatment with vitamin E and lutein protected HepG2 cells against HP-induced oxidative stress via strengthening the antioxidant system.

### 3.4. Transcriptome Data Quality Assessment of Vitamin E-, Lutein-, or HP-Treated HepG2 Cells

**Figure 4:**
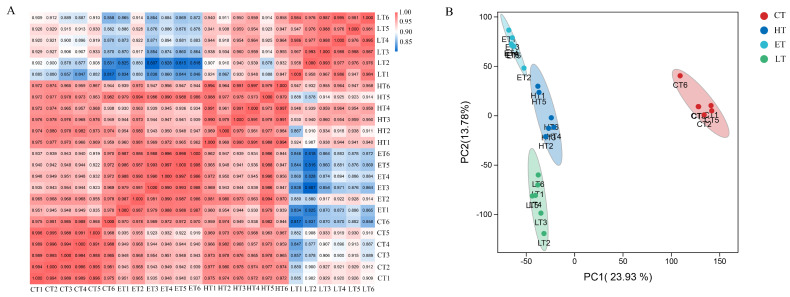
Transcriptome data evaluation of vitamin E-, lutein-, or H_2_O_2_-treated HepG2 cells. (**A**) Pearson’s correlation coefficients were calculated between samples in CT, ET, HT, and LT groups. The color from blue to red indicates the coefficients from low to high. (**B**) PCA. The dots with different colors represent different groups of samples. The percentages of the explained values of PC1 and PC2 are 23.93% and 13.78%, respectively.

To further investigate the mechanisms of how vitamin E and lutein ameliorate HP-triggered oxidative cytotoxicity in HepG2 Cells, RNA-seq was utilized to explore the changes in transcriptional level. HepG2 cells were subjected to four different treatments, including control (CT/CM), H_2_O_2_ (HT/HM), vitamin E + H_2_O_2_ (ET/EM), and lutein + H_2_O_2_ (LT/LM). A total of 168.78 Gb Clean Data with Q30 > 96.55% were acquired from 24 libraries (Table S2). Overall, 97.11–98.64% short clean reads of different samples were mapped to the reference genome (Homo_sapiens.GRCh38_release95.genome.fa), of which a total of 8391 new genes were discovered, and 2695 of them received functional annotation. Pearson’s correlation coefficients between the six replicated samples were greater than 0.94, which indicated the data were repeatable and reasonable (Figure 4A). According to principal component analysis (PCA), 23.93% and 13.78% of the total variance were explained by PC1 and PC2, respectively (Figure 4B). The four treatment groups of CT, ET, LT, and HT were separated into four clusters, suggesting that pretreatment with vitamin E or lutein elicited changes in the transcriptional program of HepG2 cells exposed to HP stress.

### 3.5. Characteristics of DEGs Profiling in Vitamin E-, Lutein-, or HP-Treated HepG2 Cells

**Figure 5:**
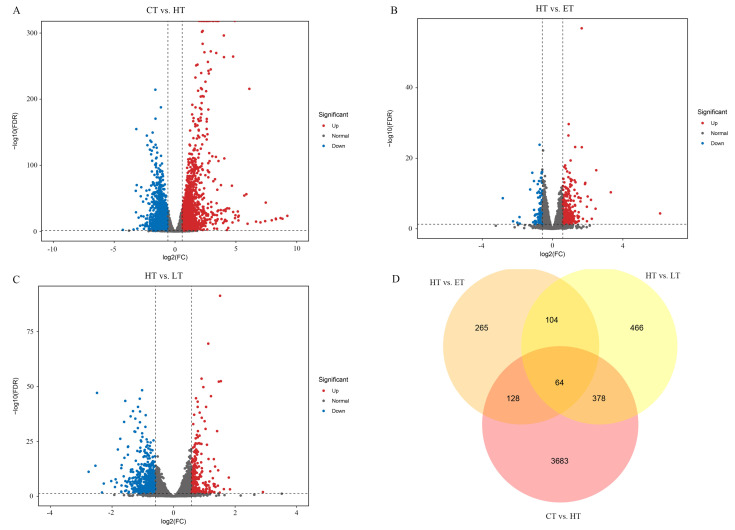
DEGs of HP-triggered stress, and vitamin or lutein treatment under oxidative stress. (**A**–**C**) Volcano plots of DEGs between comparisons of CT vs. HT, HT vs. ET, and HT vs. LT, respectively. Red and blue dots represent upregulated and downregulated genes, respectively, and gray dots indicate no significant change. (**D**) Venn diagram analysis of DEGs between three pairwise comparisons.

We performed three specific comparisons between these groups for a significative and comprehensive analysis: CT vs. HT (indicating oxidative stress induction), HT vs. ET (indicating the ameliorative effect of vitamin E against oxidative damage), and HT vs. LT (indicating the ameliorative effect of lutein against oxidative damage). As shown in Table S3, HP-triggered oxidative cytotoxicity altered the expression of 4253 genes (2151 upregulated and 2102 downregulated). Pretreatment with vitamin E modulated this response, significantly changing the expression of 561 genes (432 upregulated, 129 downregulated). In contrast, lutein pretreatment resulted in a greater number of alterations, with 263 genes upregulated and 749 genes downregulated compared to the HP treatment alone (Figure 5A–C). There are 192 common differentially expressed genes (DEGs) between the comparison of CT vs. HT and HT vs. ET, and 442 common DEGs between the comparison of CT vs. HT and HT vs. LT (Figure 5D), which are in response to vitamin E and lutein under oxidative stress, respectively. The 64 DEGs overlapped between CT vs. HT, HT vs. ET, and HT vs. LT indicated these genes were involved in both vitamin E and lutein pretreatment of HP-induced HepG2 cells. The responses of six DEGs to oxidative stress, either from this study or previous publications, underwent qRT-PCR validation, including heme oxygenase 1 (HMOX1), diamine acetyltransferase 1 (SAT1), metallothionein (MT2A, MT1G), S-phase kinase-associated protein 2 (SKP2), and cyclin-dependent kinase inhibitor 1 (CDKN1A). It indicates consistent results between the transcriptome sequencing data and the qRT-PCR results (Figure S2).

### 3.6. GO Enrichment Analysis of DEGs

**Figure 6:**
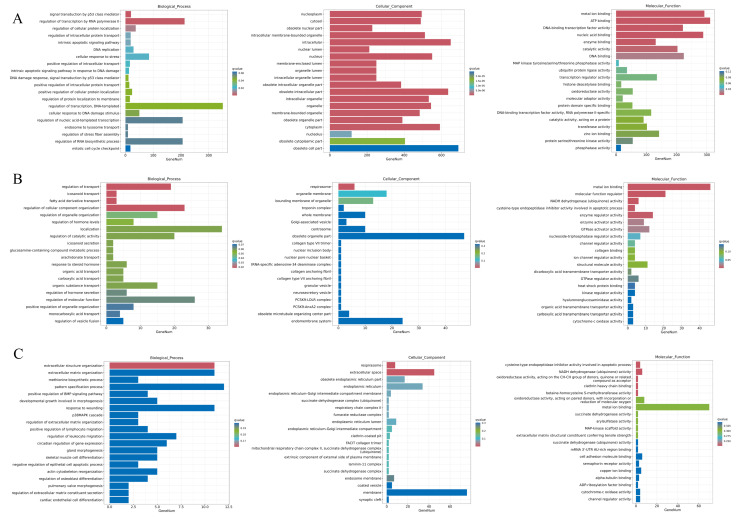
GO enrichment analysis of DEGs. Three categories of biological process, cellular component, and molecular function enrichment in CT vs. HT (**A**), HT vs. ET (**B**), and HT vs. LT (**C**). The horizontal axis represents the number of genes annotated in this entry, and the vertical axis represents each GO annotation entry. Column of color represents hypergeometric inspection q value.

GO analysis was conducted to explore the functional annotations of DEGs enrichment in three classifications: biological process, cellular component, and molecular function. In the comparison of CT vs. HT, the biological process was mainly enriched in the GO terms of signal transduction by p53 class mediator (20, 0.86%), regulation of transcription by RNA polymerase II (213, 9.12%), regulation of cellular protein localization (37, 1.58%), regulation of intracellular protein transport (19, 0.81%), and intrinsic apoptotic signaling pathway (19, 0.81%). The cellular component was mainly enriched in the GO terms of nucleoplasm (494, 19.75%), cytosol (489, 19.55%), obsolete nuclear part (228, 9.12%), intracellular membrane-bounded organelle (511, 20.43%), intracellular (650, 25.99%). The molecular function was mainly enriched in the GO terms of metal ion binding (292, 10.77%), ATP binding (311, 11.47%), DNA-binding transcription factor activity (221, 8.15%), nucleic acid binding (288, 10.62%), and enzyme binding (131, 4.83%) (Figure 6A).

For the comparison of HT vs. ET, the biological process mainly comprised DEGs associated with regulation of transport (19, 8.23%), icosanoid transport (3, 1.30%), fatty acid derivative transport (3, 1.30%), regulation of cellular component organization (23, 9.96%), and regulation of organelle organization (15, 6.49%). The cellular component mainly comprised DEGs associated with respirasome (6, 2.09%), organelle membrane (18, 6.27%), bounding membrane of organelle (13, 4.53%), troponin complex (2, 0.70%), and whole membrane (10, 3.48%). The molecular function mainly comprised DEGs associated with metal ion binding (46, 15.81%), molecular function regulator (21, 7.22%), NADH dehydrogenase (ubiquinone) activity (6, 2.06%), cysteine-type endopeptidase inhibitor activity involved in apoptotic process (4, 1.37%), and enzyme regulator activity (14, 4.81%) (Figure 6B).

For the comparison of HT vs. LT, the GO terms of extracellular structure organization (11, 2.22%), extracellular matrix organization (11, 2.22%), methionine biosynthetic process (3, 0.60%), pattern specification process (12, 2.42%), and positive regulation of BMP signaling pathway (4, 0.81%) were enriched in the classification of biological process. The GO terms of respirasome (8, 1.42%), extracellular space (45, 7.98%), obsolete endoplasmic reticulum part (17, 3.01%), endoplasmic reticulum (34, 6.03%), and endoplasmic reticulum-Golgi intermediate compartment membrane (4, 0.71%) were enriched in the category of the cellular component. The GO terms of cysteine-type endopeptidase inhibitor activity involved in the apoptotic process (4, 0.63%), NADH dehydrogenase (ubiquinone) activity (6, 0.95%), oxidoreductase activity (acting on the CH-CH group of donors, quinone or related compound as acceptor) (2, 0.32%), clathrin heavy chain binding (2, 0.32%), and betaine-homocysteine S-methyltransferase activity (2, 0.32%) were enriched in the classification of the molecular function (Figure 6C). The three comparisons revealed both overlapped and specific DEGs within enriched GO terms.

### 3.7. Metabolome Data Quality Assessment of Vitamin E-, Lutein-, or HP-Treated HepG2 Cells

**Figure 7:**
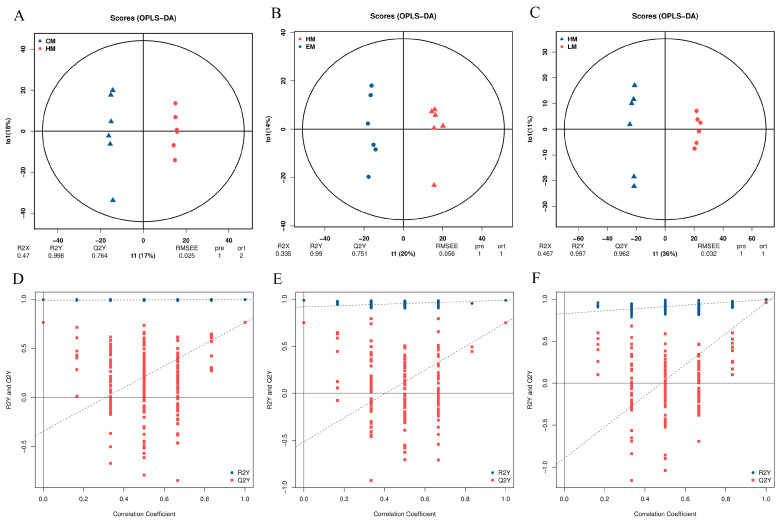
Metabolome data evaluation of vitamin E-, lutein-, or HP-treated HepG2 cells. (**A**–**C**) OPLS-DA score plots in comparisons of CM vs. HM, HM vs. EM, and HM vs. LM; (**D**–**F**) the corresponding validation plots based on 200 times permutation tests. The OPLS-DA models were valid, reliable, and stable in performance as Q2Y > 0.5 and the slope of all the blue regression lines of the Q2Y points is positive.

The metabolic accumulation changes in HepG2 cells with HP-induced stress, and with vitamin E or lutein treatment under oxidative stress were investigated based on LC-MS/MS. The accuracy and reproducibility of the metabolomic analysis were assessed by examining the total ion current (TIC) chromatograms. The QC samples demonstrated strong overlap in both peak intensity and retention time across positive and negative ion modes (Figure S3). A total of 1706 metabolites were recognized from four groups of samples (Table S4), which were further performed with OPLS-DA for variable evaluation. In comparisons of CM vs. HM, HM vs. EM, and HM vs. LM, the model explanation ratios R2X were 0.47, 0.335, and 0.467, while R2Y values were 0.998, 0.99, and 0.997, respectively. The Q2Y values indicating model prediction abilities were 0.764, 0.751, and 0.962, in CM vs. HM, HM vs. EM, and HM vs. LM (Figure 7A–C). These data suggested that all models were valid, reliable, and stable in performance. The corresponding validation was plotted following 200 times permutation tests. The validity of the OPLS-DA models was confirmed through 200 permutation tests, with the Q2Y regression line intersecting the vertical axis below zero (Figure 7D–F), demonstrating the absence of overfitting.

### 3.8. The Metabolic Pathway Analysis of Vitamin E-, Lutein-, or HP-Treated HepG2 Cells

**Figure 8:**
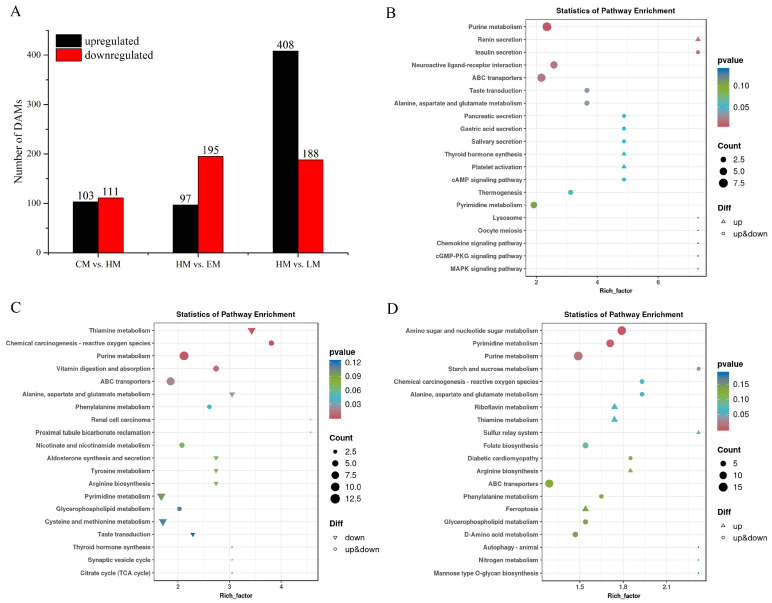
DAM statistics and KEGG pathway enrichment analyses. (**A**) Distribution of upregulated and downregulated DAMs in three comparisons. KEGG pathway enrichment in CM vs. HM (**B**), HM vs. EM (**C**), and HM vs. LM (**D**). The left vertical axis represents the KEGG pathway. The horizontal axis indicates the enrichment factor. The larger the enrichment factor is, the more significant the enrichment level of DAMs in this pathway is. A smaller *p* value indicates that the DAMs in this pathway have a more reliable significance for enrichment.

To systematically investigate differential metabolites in vitamin E-, lutein-, or HP-treated HepG2 Cells, DAMs were screened on the basis of thresholds of FC > 1, VIP ≥ 1, and *p* < 0.05. A total of 214 DAMs were identified in CM vs. HM, including 103 upregulated and 111 downregulated DAMs. A total of 292 DAMs were screened in HM vs. EM, consisting of 97 upregulated and 195 downregulated DAMs. A total of 596 DAMs were discovered in HM vs. LM, comprising 408 upregulated and 188 downregulated DAMs (Figure 8A). The distribution of DAMs across relevant metabolic pathways was assessed via KEGG enrichment analysis in HepG2 cells under different treatments. Under oxidative damage, the DAMs were mainly enriched in KEGG pathways of purine metabolism, insulin secretion, renin secretion, neuroactive ligand−receptor interaction, and ABC transporters (Figure 8B). With vitamin E ameliorative effects, the disturbed metabolic pathways mainly included thiamine metabolism, chemical carcinogenesis−reactive oxygen species, purine metabolism, vitamin digestion and absorption, and ABC transporters (Figure 8C). With lutein’s ameliorative effects, KEGG pathway analysis showed significant enrichment of amino sugar and nucleotide sugar metabolism, as well as pyrimidine metabolism, purine metabolism, starch and sucrose metabolism, and chemical carcinogenesis−reactive oxygen species (Figure 8D). These pathways give play to their potentially serving as the targeted pathways of vitamin E or lutein against oxidative damage.

## 4. Discussion

Normal physiological metabolism inevitably generates ROS from inhaled oxygen. Elevated levels of ROS in the body can result from an unhealthy external environment and/or aberrant internal metabolism [17]. Surplus ROS triggers oxidative stress, thereby causing cellular oxidative damage, which is a well-recognized risk factor of various chronic illnesses, notably cardiovascular disease and diabetes [18]. In the study, oxidative damage was induced by H_2_O_2_, a stable donor of free radicals commonly used to establish in vitro oxidative stress model. To mitigate the negative effect, the antioxidant activities of food functional factors have been paid increasing attention. Being safe, common, and easily accessible, vitamin E [19], the nutritional ingredient, and lutein [20], the bioactive substance in food, were used as representative examples to investigate the antioxidant mechanisms of food functional factors.

Treatment of HepG2 cells with HP for 6 h led to elevated levels of ROS, and showed dose-dependent effects. However, low concentrations of HP did not affect cell viability, and the cell viability began to decline until the concentration reached 0.8 mM, indicating the appearance of cell cytotoxicity. Compared with HP stress, optimal concentrations of vitamin E or lutein pretreatment demonstrated a greater increase in cell viability and decrease in ROS intensity. In addition, vitamin E had greater efficacy on ameliorating cell cytotoxicity than lutein.

As a key antioxidant enzyme, SOD protects cells by catalyzing the dismutation of superoxide radicals into H_2_O_2_, thereby scavenging harmful oxygen free radicals [21]. CAT could decompose H_2_O_2_ into innoxious H_2_O and O_2_ molecule [22]. Consistent with prior evidence that H_2_O_2_ suppresses antioxidant enzyme activities (e.g., SOD and CAT) in HepG2 cells [23], this study demonstrates that application of vitamin E and lutein significantly (*p* < 0.05) attenuated this H_2_O_2_-induced inhibition. GSH, an antioxidant peptide synthesized endogenously in the liver, performs a crucial role in protecting this organ resulting from its high susceptibility to ROS [24,25]. The GSH content was also significantly elevated by treatment with vitamin E or lutein under oxidative stress, and GSH content recovered at higher levels via vitamin E pretreatment compared to lutein. Our findings demonstrated that both vitamin E and lutein alleviated oxidative cytotoxicity in HepG2 cells by improving the antioxidant system, and vitamin E exhibited superior efficacy.

This study sought to systematically assess and compare the ameliorative effects of vitamin E and lutein on HP-triggered oxidative cytotoxicity in HepG2 cells on the basis of integrated transcriptomic and metabolomic profiling. Transcriptome studies suggested that the repeatability of six replications in each treatment was good. And the groups of CT, HT, ET, and LT were separated from each other on the basis of PCA, suggesting that the gene expression profile of HepG2 cells in response to oxidative stress was altered by pretreatment with either vitamin E or lutein. A total of 28,399 genes were found in the study, of which 5088 were defined as DEGs among the three comparisons of CT vs. HT, HT vs. ET, and HT vs. LT, and 64 DEGs were common to them. A total of 128 DEGs specifically responded to vitamin E under HP-induced stress. And a total of 378 DEGs particularly responded to lutein under oxidative stress.

GO analysis demonstrated that HP treatment significantly activated crucial biological processes, cellular components, and molecular functions, including signal transduction by p53 class mediator, intrinsic apoptotic signaling pathway, metal ion binding, ATP binding, and DNA-binding transcription factor activity. p53, as a tumor suppressor gene, is the central component of signaling pathways in a complex network. Thousands of unique genes were regulated by p53-induced organism protection [26]. In a previous study, octyl gallate and gallic acid inhibited breast cancer progression through activating the intrinsic apoptotic signaling pathway, coupled with the upregulation of antioxidant enzymes SOD and CAT [27]. Metallothioneins (MTs) perform a crucial role in cellular defense, utilizing their cysteine-rich structure to bind heavy metal ions and mitigate an array of stress responses including oxidative stress [28]. The expression of metallothionein (MT2A, MT1G) was upregulated with HP treatment. The results indicated that MT-associated molecular function in HepG2 cells was activated by HP. The ATP-binding cassette transporter subfamily G member 2 (ABCG2) mediates protection from cardiac hypertrophy and heart failure under pressure overload via activation of antioxidant response pathways [29]. DNA-binding transcription factor activity is closely related to oxidative response, and is well-documented in the scientific literature. For example, NF-κB, a redox-sensitive transcription factor central to the cellular oxidative stress response, demonstrates clinical relevance as the DNA-binding activity of its p65 subunit shows a significant correlation with systemic levels of antioxidants in breast carcinoma patients [30]. Transport-related GO terms (regulation of transport, icosanoid transport, fatty acid derivative transport), enzyme-related GO terms (NADH dehydrogenase (ubiquinone) activity, enzyme regulator activity), oxidative stress-related GO terms (metal ion binding), and apoptosis-related GO terms (cysteine-type endopeptidase inhibitor activity involved in apoptotic process) were meaningfully enriched in vitamin E-pretreated HepG2 cells under oxidative stress. One of the antioxidant mechanisms of mitochondria-targeted cationic plastoquinone derivatives was mild uncoupling by fatty acid cycling that suppresses the formation of ROS [31]. The generation of ROS following endosulfan treatment is linked to a decline in mitochondrial NADH dehydrogenase activity within rat brain [32]. Extracellular organization-related GO terms (extracellular structure organization, extracellular matrix organization), biological process-related GO terms (methionine biosynthetic process, pattern specification process, positive regulation of BMP signaling pathway), apoptosis-related GO terms (cysteine-type endopeptidase inhibitor activity involved in apoptotic process), and enzyme-related GO terms (NADH dehydrogenase (ubiquinone) activity, oxidoreductase activity, acting on the CH-CH group of donors, quinone or related compound as acceptor, betaine-homocysteine S-methyltransferase activity) were meaningfully enriched in lutein-pretreated HepG2 cells under oxidative stress. Müller cells are responsible for multiple critical physiological functions in the retina, including regulating extracellular composition, contributing to the blood–retina barrier structure, and exerting antioxidant effects. Dysregulation of these functions influences the severity of diabetic retinopathy [33]. Thus, application of lutein holds promise as a novel therapeutic strategy for this disease. Methionine, as a sulfur metabolite, has antioxidant potential and can maintain liver health [34]. Genetic suppression of the methionine metabolism genes MAT2A or AHCY leads to the induction of oxidative stress [35]. In the present study, MT2A and MT1G expression were further elevated with lutein pretreatment compared to HP stress, suggesting lutein ameliorates oxidative stress partly owing to metallothionein. Vardenafil counters H_2_O_2_-induced downregulation of BMP-binding endothelial regulator protein, thereby protecting endothelial cells from oxidative damage via a BMP pathway-linked mechanism [36]. Our study demonstrated the protective function of lutein in HP-induced HepG2 cell injury is also partly mediated through positive regulation of BMP signaling pathway. Quinone oxidoreductase 1 (NQO1) is a cytoprotective antioxidant enzyme that detoxifies quinones through a two-electron reduction pathway, which is a critical step for their bioreductive activation [37]. Lutein activated oxidoreductase activity in the HepG2 cells under oxidative stress, suggesting its role in ameliorating endogenous oxidative damage and maintaining health.

In this study, non-targeted metabolomics showed excellent machine stability with negligible technical variation. And the OPLS-DA models demonstrated stable, reliable, and excellent performance in variable assessment. Under HP-induced oxidative stress, purine metabolism, renin secretion, insulin secretion, and ABC transporters were meaningfully enriched KEGG pathways. The precise regulation of purine metabolism is a critical node in the oxidative stress response. An imbalance in purine metabolism, specifically between salvage and de novo synthesis, can exacerbate oxidative stress by generating damaging ROS [38]. In vitro experiments demonstrated that angiotensin II triggered superoxide generation in mesangial cells, a key mechanism driving oxidative stress that can be alleviated by inhibiting the renin–angiotensin system (RAS) [39]. Obesity is a complex metabolic disease, and is a crucial risk factor for type 2 diabetes. RAS-mediated oxidative stress acts as a critical pathogenic bridge, connecting obesity and insulin resistance [40]. In lung cancer cells, ABC transporter inhibitors elevated intracellular ROS levels and augmented the cytotoxicity of chemicals, thereby underscoring the essential detoxification role these transporters play [41]. In vitamin E pretreatment under oxidative stress, thiamine metabolism, chemical carcinogenesis−reactive oxygen species, purine metabolism, vitamin digestion and absorption, and ABC transporters were enriched KEGG pathways. As a key vitamin, thiamine acts as a central cofactor in energy, lipid, and amino acid metabolism across all organisms, and also performs a crucial role in stress response. Under H_2_O_2_-induced oxidative stress, fission cells cultured in a thiamine-rich medium upregulated genes involved in thiamine biosynthesis and transport [42]. A previous study indicated that the event chemical carcinogenesis-reactive oxygen species is also implicated in the cadmium-induced oxidative stress [43]. As an essential nutrient and key antioxidant, vitamin E (alpha-tocopherol) relies on oral intake, absorption, and alpha-tocopherol transfer protein (alpha-TTP)-facilitated lipoprotein transfer to maintain its plasma levels. Furthermore, the localization of alpha-TTP in cerebellar Purkinje cells is observed in patients experiencing vitamin E deficiency or diseases characterized by oxidative damage [44]. In lutein pretreatment under oxidative stress, amino sugar and nucleotide sugar metabolism, pyrimidine metabolism, purine metabolism, starch and sucrose metabolism, and chemical carcinogenesis−reactive oxygen species were enriched KEGG pathways. Nucleotide sugar transporters (NSTs) are implicated in the oxidative damage response, as evidenced by the upregulation of the EiNST5 transcript during in vitro encystation and oxidative stress in *Entamoeba invadens*, and likewise by the similar upregulation of EhNST3 under oxidative stress in *Entamoeba histolytica* [45]. Functional enrichment analysis also identified that amino sugar and nucleotide sugar metabolism is one of the most enriched KEGG pathways in *Candida albicans* under H_2_O_2_-induced oxidative stress [46]. In stomach adenocarcinoma, GPX3-driven oxidative stress mediates alterations in pyrimidine metabolism through the AMPK/mTOR signaling pathway [47]. Sucrose metabolism functions as a vital regulator of the stress response by producing a range of sugar-derived metabolites that modulate the expression of oxidative defense components, including microRNAs, transcription factors, and other genes [48]. Vitamin E and lutein activated both common and distinct KEGG pathways.

## 5. Conclusions

This study systematically evaluated and compared the cytoprotective potential and antioxidant of vitamin E and lutein under HP-triggered oxidative cytotoxicity in HepG2 cells employing integrated transcriptomics and metabolomics, along with physiology and biochemistry. Exposure to H_2_O_2_ downregulated cell viability and upregulated ROS level in a dose-dependent manner. An amount of 0.8 mM H_2_O_2_ led to cellular oxidative damage and further downregulated antioxidant enzyme SOD and CAT activities, and antioxidant GSH content. Pretreatment with concentrations of 10 μΜ vitamin E or 80 μΜ lutein effectively ameliorated cell cytotoxicity, as evidenced by recovering cell viability and ROS level and balancing the redox system. And vitamin E had greater efficacy than lutein. Transcriptome profiling showed that vitamin E mainly activated transport-related, enzyme-related, oxidative stress-related, and apoptosis-related GO terms to ameliorate oxidative stress, whereas lutein mainly activated extracellular organization-related and biological process-related GO terms, in addition to apoptosis-related and enzyme-related GO terms for oxidation resistance. Metabolome profiling indicated metabolites were markedly enriched in KEGG pathways of thiamine metabolism, chemical carcinogenesis−reactive oxygen species, purine metabolism, vitamin digestion and absorption, and ABC transporters with vitamin E pretreatment under oxidative stress. In contrast, metabolites were markedly enriched in KEGG pathways of amino sugar and nucleotide sugar metabolism, pyrimidine metabolism, and starch and sucrose metabolism, in addition to purine metabolism and chemical carcinogenesis−reactive oxygen species, with lutein pretreatment under oxidative stress. This study is the first to comprehensively analyze and compare the molecular mechanisms of vitamin E and lutein in HepG2 cells under H_2_O_2_-induced stress condition.

## Data Availability

The data supporting the findings of this study are available from the corresponding author upon reasonable request.

## Supplementary Materials

The following supporting information can be downloaded at: https://www.mdpi.com/article/10.3390/cells14242020/s1, Figure S1: Cytotoxicity of vitamin E and lutein in HepG2; HepG2 cells were treated with 1–40 μΜ vitamin E or 20–160 μM lutein for 12 h. Cell viability analyzed by CCK-8 assay; Figure S2: qRT-PCR validation of six DEGs with vitamin E and lutein treatment and under HP-induced stress; Figure S3: TIC chromatograms across positive and negative ion modes; Table S1: The primers used in the study; Table S2: Summary statistics of transcriptome sequencing; Table S3: Information of identified genes; Table S4: Information of identified metabolites.

## Abbreviations

The following abbreviations are used in this manuscript:

ROS	Reactive oxygen species
HP	Hydrogen peroxide
DMEM	Dulbecco’s Modified Eagle’s Medium
CCK-8	Cell Counting Kit-8
DCFH-DA	2′,7′-dichlorofluorescein diacetate
SOD	Superoxide dismutase
CAT	Catalase
GSH	Glutathione
WST-1	Water-soluble tetrazolium salt
GO	Gene Ontology
FPKM	Fragments per kilobase of transcript per million fragments mapped
FDR	False discovery rate
DEGs	Differentially expressed genes
qRT-PCR	Quantitative real-time polymerase chain reaction
QC	Quality control
UPLC-QTOF-MS/MS	Ultra-high Performance Liquid Chromatography-Quadrupole Time-of-flight-Tandem Mass Spectroscopy
KEGG	Kyoto Encyclopedia of Genes and Genomes
FC	Fold change
OPLS-DA	Orthogonal partial least squares-discriminant analysis
VIP	Variable importance in projection
ANOVA	One-way analysis of variance
PCA	Principal component analysis
TIC	Total ion current
MTs	Metallothioneins
NSTs	Nucleotide sugar transporters

## References

[1] Li Z.Q., Gao Y.T., Zhao C.F., An R., Wu Y.L., Huang Z.P., Ma P., Yang X., She R., Yang X.Y. (2025). Antioxidant and anti-inflammatory function of walnut green husk aqueous extract (WNGH-AE) on human hepatocellular carcinoma cells (HepG2) treated with t-BHP. PLoS ONE.

[2] Vakifahmetoglu-Norberg H., Ouchida A.T., Norberg E. (2017). The role of mitochondria in metabolism and cell death. Biochem. Biophys. Res. Commun..

[3] Albertolle M.E., Guengerich F.P. (2018). The relationships between cytochromes P450 and H_2_O_2_: Production, reaction, and inhibition. J. Inorg. Biochem..

[4] Uchida Y., Ferdousi F., Takahashi S., Isoda H. (2024). Comprehensive transcriptome profiling of antioxidant activities by glutathione in human HepG2 cells. Molecules.

[5] Sunthrarak C., Posridee K., Noisa P., Shim S., Thaiudom S., Oonsivilai A., Oonsivilai R. (2025). Synergistic antioxidant and cytoprotective effects of Thunbergia laurifolia Lindl and Zingiber officinale extracts against PM2.5-induced oxidative stress in A549 and HepG2 cells. Foods.

[6] Ni C., Zhou W., Yu M., Li X., Li J., Cui Y., Cui W. (2023). Vitamin E treatment improves the antioxidant capacity of patients receiving dialysis: A systematic review and meta-analysis. Mol. Nutr. Food Res..

[7] Sepidarkish M., Morvaridzadeh M., Akbari-Fakhrabadi M., Almasi-Hashiani A., Rezaeinejad M., Heshmati J. (2019). Effect of omega-3 fatty acid plus vitamin E Co-Supplementation on lipid profile: A systematic review and meta-analysis. Diabetes Metab. Syndr..

[8] Enogieru A.B., Idemudia O.U. (2025). Antioxidant activity and upregulation of BDNF in lead acetate–exposed rats following pretreatment with vitamin E. Comp. Clin. Pathol..

[9] Kavalappa Y.P., Gopal S.S., Ponesakki G. (2021). Lutein inhibits breast cancer cell growth by suppressing antioxidant and cell survival signals and induces apoptosis. J. Cell. Physiol..

[10] Armoza A., Haim Y., Basiri A., Wolak T., Paran E. (2013). Tomato extract and the carotenoids lycopene and lutein improve endothelial function and attenuate inflammatory NF-κB signaling in endothelial cells. J. Hypertens..

[11] Gong M., Lu H., Li L., Feng M., Zou Z. (2023). Integration of transcriptomics and metabonomics revealed the protective effects of hemp seed oil against methionine-choline-deficient diet-induced non-alcoholic steatohepatitis in mice. Food Funct..

[12] Jayaraman V., Arumugam M.K., Balachandran S., Boopathy L., Arumugam S., Srirangaramasamy J., Devanesan S., Suresh A., Sampath S. (2025). Anticancer effects of monacolin X against human liver cancer cell line: Exploring the apoptosis using AO/EB and DCFHDA fluorescent staining. Luminescence.

[13] Peskin A.V., Winterbourn C.C. (2000). A microtiter plate assay for superoxide dismutase using a water-soluble tetrazolium salt (WST-1). Clin. Chim. Acta.

[14] Ozmen B., Ozmen D., Erkin E., Güner I., Habif S., Bayindir O. (2002). Lens superoxide dismutase and catalase activities in diabetic cataract. Clin. Biochem..

[15] Lv H., Xu J., Bo T., Wang W. (2020). Comparative transcriptome analysis uncovers roles of hydrogen sulfide for alleviating cadmium toxicity in *Tetrahymena thermophila*. BMC Genom..

[16] Livak K.J., Schmittgen T.D. (2001). Analysis of relative gene expression data using real-time quantitative PCR and the 2^−ΔΔCT^ method. Methods.

[17] Ruan J., Chen J., Zeng J., Yang Z., Wang C., Hong Z., Zuo Z. (2019). The protective effects of Nile tilapia (*Oreochromis niloticus*) scale collagen hydrolysate against oxidative stress induced by tributyltin in HepG2 cells. Environ. Sci. Pollut. Res. Int..

[18] Guha S., Majumder K. (2019). Structural-features of food-derived bioactive peptides with anti-inflammatory activity: A brief review. J. Food Biochem..

[19] Pérez-Lamela C., Torrado-Agrasar A.M. (2025). Effects of high-pressure processing (HPP) on antioxidant vitamins (A, C, and E) and antioxidant activity in fruit and vegetable preparations: A review. Appl. Sci..

[20] Ma Y., Zhang S., Zhou X., Wang X., Shen W., Ge X. (2025). Preparation of the co-delivery system polymersome for blueberry anthocyanins/lutein and study on its antioxidant and transdermal absorption properties. Food Biosci..

[21] Jiang Z., Wang W., Guo C. (2017). Tetrahydroxy stilbene glucoside ameliorates H_2_O_2_-induced human brain microvascular endothelial cell dysfunction invitro by inhibiting oxidative stress and inflammatory responses. Mol. Med. Rep..

[22] Lv H., Guo S., Wu Z., Nan X., Zhu M., Mao K. (2024). Postharvest quality and metabolism changes of daylily flower buds treated with hydrogen sulfide during storage. Postharvest Biol. Technol..

[23] Gao W., Zhao C., Shang X., Li B., Guo J., Wang J., Wu B., Fu Y. (2025). Ameliorative effects of raisin polyphenol extract on oxidative stress and aging in vitro and in vivo via regulation of Sirt1–Nrf2 signaling pathway. Foods.

[24] Marí M., de Gregorio E., de Dios C., Roca-Agujetas V., Cucarull B., Tutusaus A., Morales A., Colell A. (2020). Mitochondrial glutathione: Recent insights and role in disease. Antioxidants.

[25] Mello T., Zanieri F., Ceni E., Galli A. (2016). Oxidative stress in the healthy and wounded hepatocyte: A cellular organelles perspective. Oxid. Med. Cell. Longev..

[26] Sun Y. (2006). p53 and its downstream proteins as molecular targets of cancer. Mol. Carcinog..

[27] Vijayalakshmi P., Indu S., Ireen C., Manjunathan R., Rajalakshmi M. (2023). Octyl gallate and gallic acid isolated from Terminalia bellirica circumvent breast cancer progression by enhancing the intrinsic apoptotic signaling pathway and elevating the levels of anti-oxidant enzymes. Appl. Biochem. Biotechnol..

[28] Bakiu R., Pacchini S., Piva E., Schumann S., Tolomeo A.M., Ferro D., Irato P., Santovito G. (2022). Metallothionein expression as a physiological response against metal toxicity in the striped rockcod *Trematomus hansoni*. Int. J. Mol. Sci..

[29] Higashikuni Y., Sainz J., Nakamura K., Takaoka M., Enomoto S., Iwata H., Tanaka K., Sahara M., Hirata Y., Nagai R. (2012). The ATP-binding cassette transporter ABCG2 protects against pressure overload-induced cardiac hypertrophy and heart failure by promoting angiogenesis and antioxidant response. Arterioscler. Thromb. Vasc. Biol..

[30] Pande D., Karki K., Negi R., Khanna S., Khanna R.S., Khanna H.D. (2013). NF-κB p65 subunit DNA-binding activity: Association with depleted antioxidant levels in breast carcinoma patients. Cell Biochem. Biophys..

[31] Skulachev V.P., Antonenko Y.N., Cherepanov D.A., Chernyak B.V., Izyumov D.S., Khailova L.S., Klishin S.S., Korshunova G.A., Lyamzaev K.G., Pletjushkina O.Y. (2010). Prevention of cardiolipin oxidation and fatty acid cycling as two antioxidant mechanisms of cationic derivatives of plastoquinone (SkQs). Biochim. Biophys. Acta.

[32] Murali M., Shivanandappa T. (2022). Endosulfan causes oxidative stress in the liver and brain that involves inhibition of NADH dehydrogenase and altered antioxidant enzyme status in rat. Ecotoxicol. Environ. Saf..

[33] Carpi-Santos R., de Melo Reis R.A., Gomes F.C.A., Calaza K.C. (2022). Contribution of Müller cells in the diabetic retinopathy development: Focus on oxidative stress and inflammation. Antioxidants.

[34] Ma G., Ayalew H., Mahmood T., Mercier Y., Wang J., Lin J., Wu S., Qiu K., Qi G., Zhang H. (2024). Methionine and vitamin E supplementation improve production performance, antioxidant potential, and liver health in aged laying hens. Poult. Sci..

[35] Rowland E.C., D’Antuono M., Jermakowicz A.M., Ayad N.G. (2025). Methionine cycle inhibition disrupts antioxidant metabolism and reduces glioblastoma cell survival. J. Biol. Chem..

[36] Mao F., Han B., Jiang D., Zhang X., Pang T., Fan Y. (2020). The Phosphodiesterase-5 inhibitor vardenafil improves the activation of BMP signaling in response to hydrogen peroxide. Cardiovasc. Drugs Ther..

[37] Rashid M.H., Babu D., Siraki A.G. (2021). Interactions of the antioxidant enzymes NAD(P)H: Quinone oxidoreductase 1 (NQO1) and NRH: Quinone oxidoreductase 2 (NQO2) with pharmacological agents, endogenous biochemicals and environmental contaminants. Chem. Biol. Interact..

[38] Tian R., Yang C., Chai S., Guo H., Seim I., Yang G. (2022). Evolutionary impacts of purine metabolism genes on mammalian oxidative stress adaptation. Zool. Res..

[39] Fanelli C., Zatz R. (2011). Linking oxidative stress, the renin-angiotensin system, and hypertension. Hypertension.

[40] Ramalingam L., Menikdiwela K., LeMieux M., Dufour J.M., Kaur G., Kalupahana N., Moustaid-Moussa N. (2017). The renin angiotensin system, oxidative stress and mitochondrial function in obesity and insulin resistance. Biochim. Biophys. Acta Mol. Basis Dis..

[41] Yuan T., Hu J., Zhu X., Yin H., Yin J. (2022). Oxidative stress-mediated up-regulation of ABC transporters in lung cancer cells. J. Biochem. Mol. Toxicol..

[42] Kartal B., Akcay A., Palabiyik B. (2018). Oxidative stress upregulates the transcription of genes involved in thiamine metabolism. Turk. J. Biol..

[43] Mei Z., Yang J., Zhao Y., Li W., Li R., Liu D., Lu H., He Z., Gu S. (2025). Comparative neurotoxic effects and mechanism of cadmium chloride and cadmium sulfate in neuronal cells. Environ. Int..

[44] Copp R.P., Wisniewski T., Hentati F., Larnaout A., Hamida M.B., Kayden H.J. (1999). Localization of α-tocopherol transfer protein in the brains of patients with ataxia with vitamin E deficiency and other oxidative stress related neurodegenerative disorders. Brain Res..

[45] Nayak S., Ghosh S.K. (2019). Nucleotide sugar transporters of *Entamoeba histolytica* and *Entamoeba invadens* involved in chitin synthesis. Mol. Biochem. Parasitol..

[46] Cui Y., Wang D., Nobile C.J., Dong D., Ni Q., Su T., Jiang C., Peng Y. (2024). Systematic identification and characterization of five transcription factors mediating the oxidative stress response in *Candida albicans*. Microb. Pathog..

[47] Zhang Y., Yang Y., Kuang S., Zhang Y., Qin H., Xie J. (2024). GPX3-mediated oxidative stress affects pyrimidine metabolism levels in stomach adenocarcinoma via the AMPK/mTOR pathway. Int. J. Clin. Pract..

[48] Ruan Y. (2014). Sucrose metabolism: Gateway to diverse carbon use and sugar signaling. Annu. Rev. Plant Biol..

