# Supplementary figures and images for: Ameliorative Effects of Vitamin E and Lutein on Hydrogen Peroxide-Triggered Oxidative Cytotoxicity via Combined Transcriptome and Metabolome Analysis
Source: Cells. 2025 Dec 18;14(24):2020. doi: 10.3390/cells14242020 (PMC12731873; doi:10.3390/cells14242020)

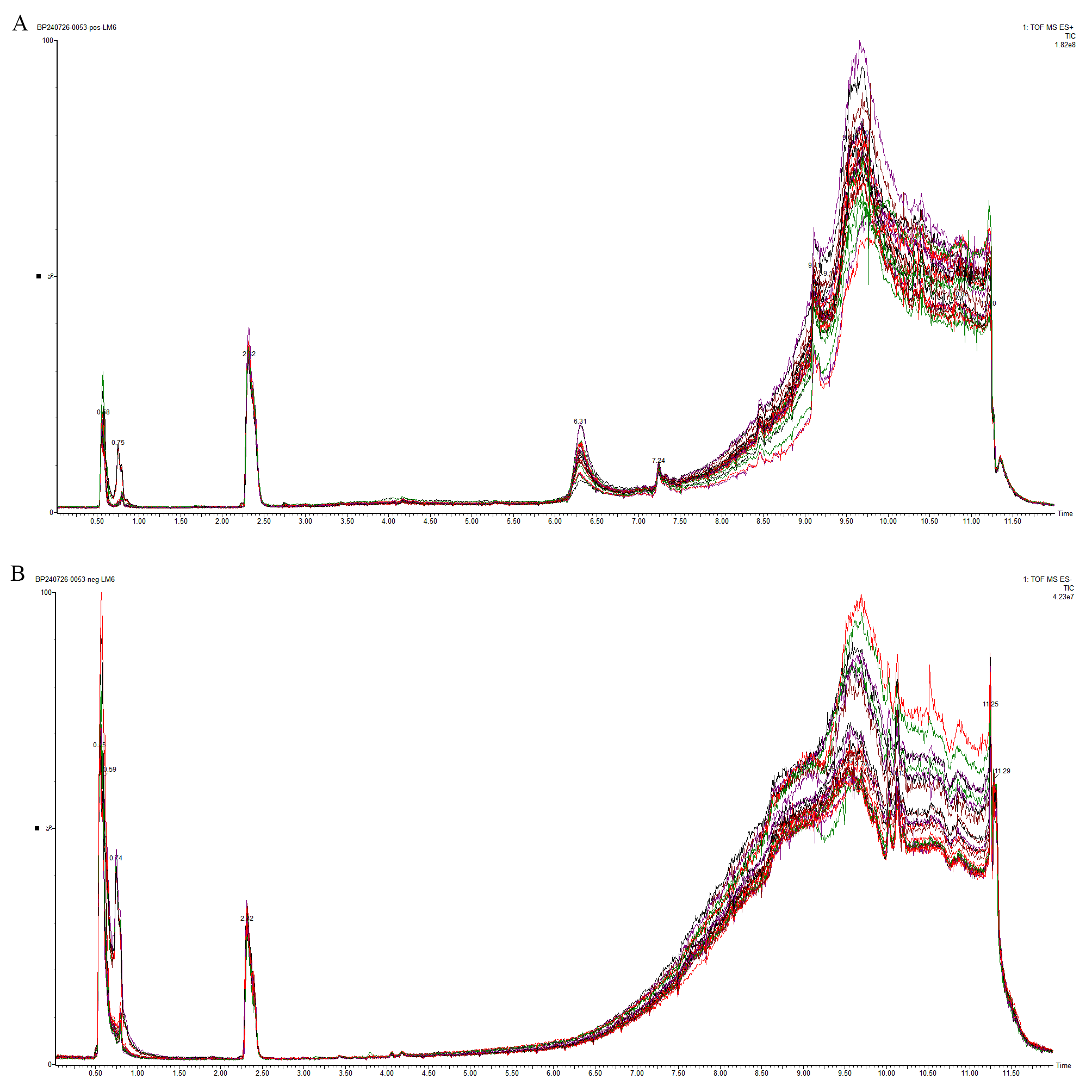

Supplement: Supplementary file 1 [file cells-14-02020-s001.zip › figure-S3.tif]

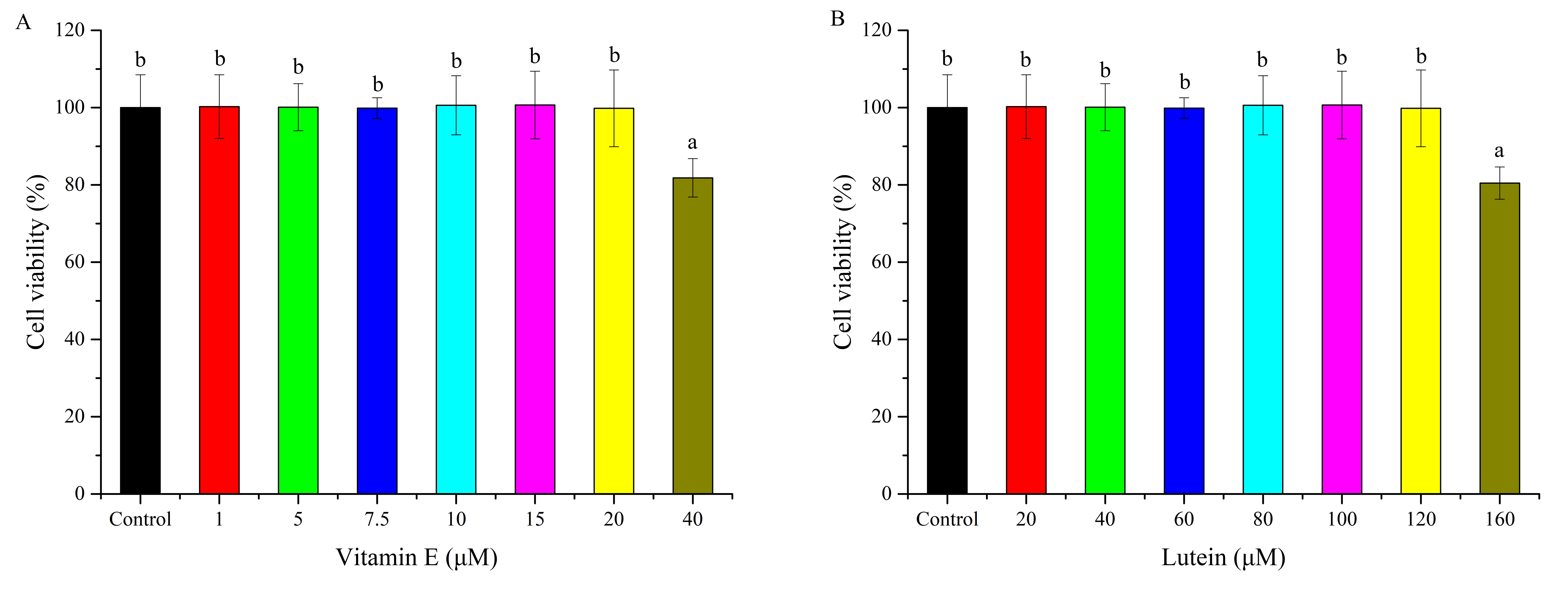

Supplement: Supplementary file 1 [file cells-14-02020-s001.zip › figure-S1.tif]

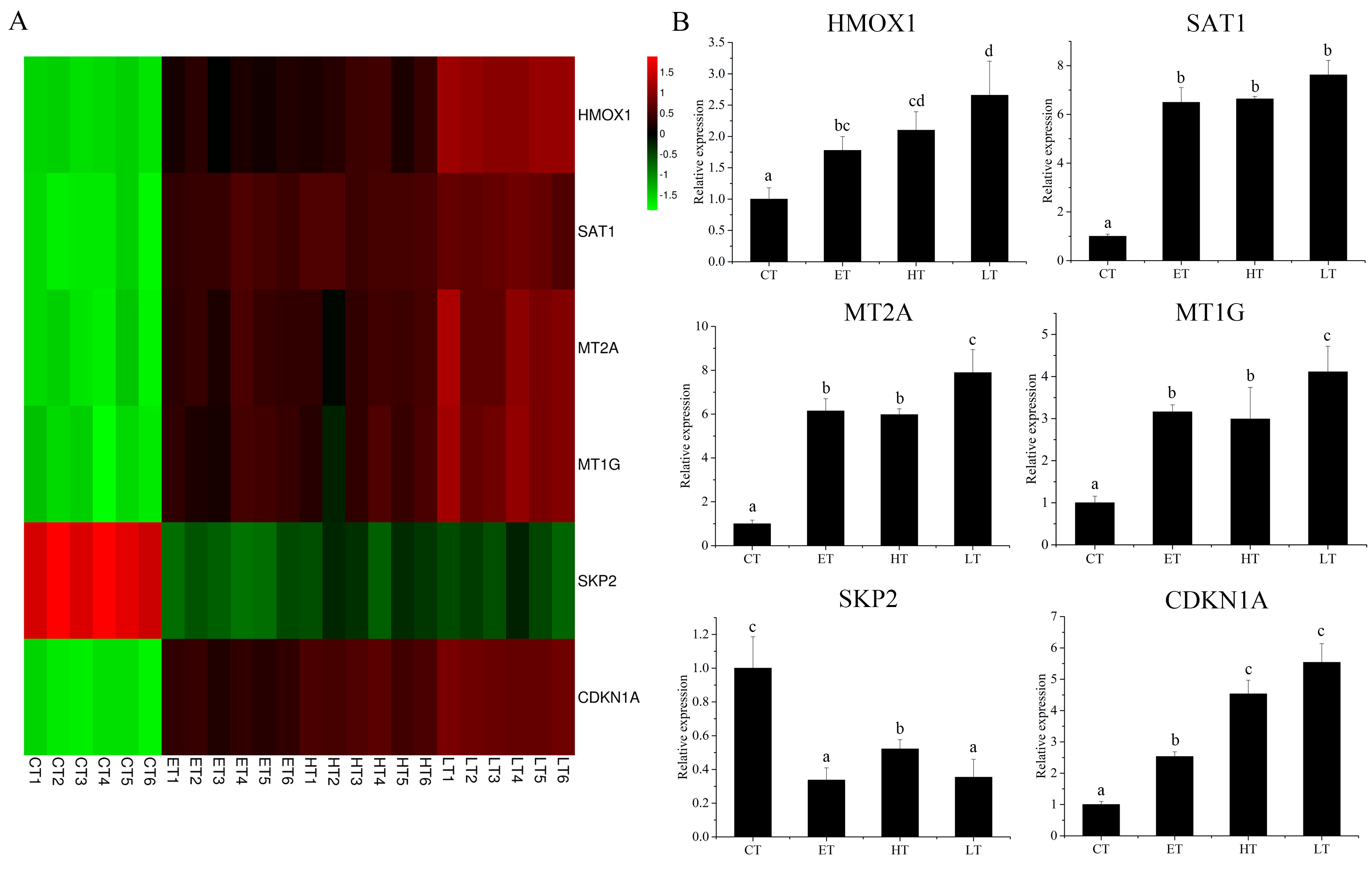

Supplement: Supplementary file 1 [file cells-14-02020-s001.zip › figure-S2.tif]
